# Supplementary figures and images for: Association of IL18 genetic polymorphisms with Chagas disease in Latin American populations
Source: PLoS Negl Trop Dis. 2019 Nov 21;13(11):e0007859. doi: 10.1371/journal.pntd.0007859 (PMC6894881; doi:10.1371/journal.pntd.0007859)

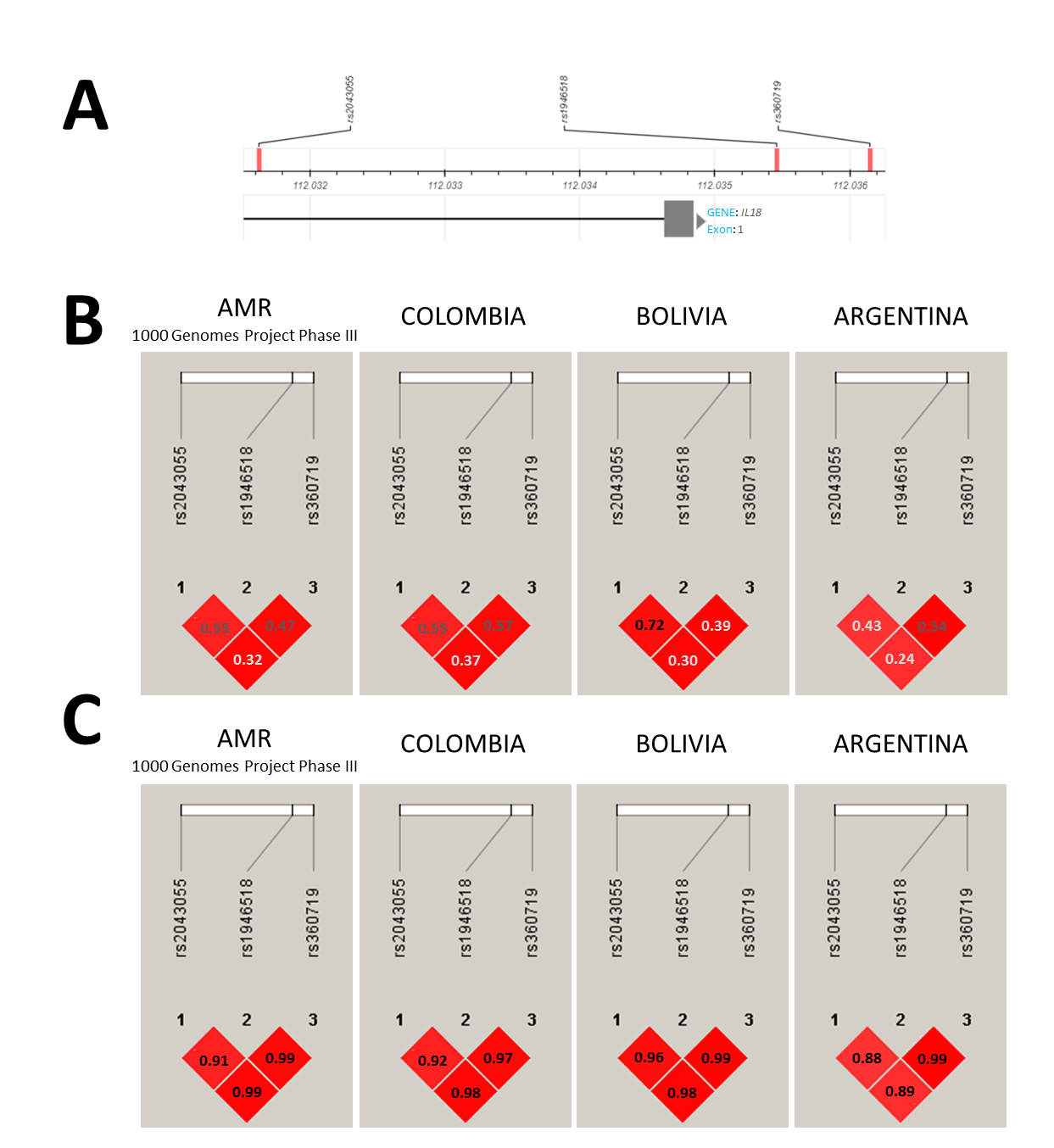

Supplement: S1 Fig — Location of IL18 rs2043055, rs1946518 and rs360719 within the gen (A). R2 (B) and Linkage disequilibrium D’ (C) plots estimated by using expectation maximization algorithmin Haploview V4.2. in Americans (AMR) from 1000 Genomes Project Phase III and in Colombian, Bolivian and Argentinian cohorts. (TIF) [file pntd.0007859.s001.tif]
