# Supplementary material for: Association of IL18 genetic polymorphisms with Chagas disease in Latin American populations
Source: PLoS Negl Trop Dis. 2019 Nov 21;13(11):e0007859. doi: 10.1371/journal.pntd.0007859 (PMC6894881; doi:10.1371/journal.pntd.0007859)
Supplement: S1 Table — Statistical power calculation of the candidate gene study (A) and the meta-analysis (B) considering the allele frequencies for each SNP, the prevalence of the disease in each country with three different OR. (DOCX) [file pntd.0007859.s002.docx]

**S1-A Table. Statistical power calculation considering different effect sizes: Candidate gene approach**

S1-A1 Table

|  | |  | *Argentina* | |
| --- | --- | --- | --- | --- |
| SNP | MAF | OR | *T. cruzi* infection (272/78)* | Chronic Chagas cardiomyopathy (182/90)* |
| rs2043055 | 41% | OR=1.60 | 84% | 80% |
|  |  | OR=1.50 | 72% | 67% |
|  |  | OR=1.40 | 56% | 51% |
|  |  | OR=1.30 | 38% | 34% |
| rs1946518 | 49% | OR=1.60 | 83% | 80% |
|  |  | OR=1.50 | 72% | 66% |
|  |  | OR=1.40 | 56% | 51% |
|  |  | OR=1.30 | 38% | 34% |
| rs360719 | 32% | OR=1.60 | 83% | 77% |
|  |  | OR=1.50 | 70% | 64% |
|  |  | OR=1.40 | 54% | 48% |
|  |  | OR=1.30 | 35% | 32% |

The estimation was performed considering a Chagas disease prevalence of 3.6% in Argentina [1]. MAF: minor allele frequency.

The allele frequencies used were those described for the Americans sub-populations of the 1000 genomes phase III project (<http://www.1000genomes.org>).

S1-A2 Table

|  |  |  | *Colombia* | |
| --- | --- | --- | --- | --- |
| SNP | MAF | OR | *T. cruzi* infection (937/640)* | Chronic Chagas cardiomyopathy (576/361)* |
| rs2043055 | 41% | OR=1.40 | 100% | 95% |
|  |  | OR=1.30 | 96% | 80% |
|  |  | OR=1.20 | 72% | 49% |
|  |  | OR=1.10 | 26% | 17% |
| rs1946518 | 49% | OR=1.40 | 100% | 95% |
|  |  | OR=1.30 | 96% | 80% |
|  |  | OR=1.20 | 72% | 50% |
|  |  | OR=1.10 | 27% | 18% |
| rs360719 | 32% | OR=1.40 | 100% | 94% |
|  |  | OR=1.30 | 94% | 77% |
|  |  | OR=1.20 | 68% | 46% |
|  |  | OR=1.10 | 24% | 16% |

The estimation was performed considering a Chagas disease prevalence of 1.44% in Colombia [1]. MAF: minor allele frequency.

The allele frequencies used were those described for the Americans sub-populations of the 1000 genomes phase III project (<http://www.1000genomes.org>).

S1-A3 Table

|  |  |  | *Bolivia* |
| --- | --- | --- | --- |
| SNP | MAF | OR | Chronic Chagas cardiomyopathy (100/530)* |
| rs2043055 | 41% | OR=1.60 | 90% |
|  |  | OR=1.50 | 79% |
|  |  | OR=1.40 | 63% |
|  |  | OR=1.30 | 43% |
| rs1946518 | 49% | OR=1.60 | 90% |
|  |  | OR=1.50 | 80% |
|  |  | OR=1.40 | 65% |
|  |  | OR=1.30 | 44% |
| rs360719 | 32% | OR=1.60 | 87% |
|  |  | OR=1.50 | 76% |
|  |  | OR=1.40 | 59% |
|  |  | OR=1.30 | 40% |

The estimation was performed considering a Chagas disease prevalence of 6.1% in Bolivia [1].

MAF: minor allele frequency.

The allele frequencies used were those described for the Americans sub-populations of the 1000 genomes phase III project (<http://www.1000genomes.org>).

S1-A4 Table

|  |  |  | *Brazil* |
| --- | --- | --- | --- |
| SNP | MAF | OR | Chronic Chagas cardiomyopathy (849/202)** |
| rs2043055 | 41% | OR=1.60 | 99% |
|  |  | OR=1.50 | 96% |
|  |  | OR=1.40 | 88% |
|  |  | OR=1.30 | 68% |

The estimation was performed considering a Chagas disease prevalence of 2.4% in Brazil [2].

MAF: minor allele frequency.

The allele frequencies used were those described for the Americans sub-populations of the 1000 genomes phase III project (<http://www.1000genomes.org>).

**S1-B Table. Statistical power calculation considering different effect sizes: Meta-analysis**

S1-B1 Table

|  |  |  | | *Argentina, Colombia* |
| --- | --- | --- | --- | --- |
| SNP | MAF | | OR | *T. cruzi* Infection (n= 1209/718)* |
| rs2043055 | 41% | | OR=1.30 | 98% |
|  |  |  | OR=1.20 | 80% |
|  |  |  | OR=1.10 | 31% |
| rs1946518 | 49% | | OR=1.30 | 98% |
|  |  |  | OR=1.20 | 80% |
|  |  |  | OR=1.10 | 31% |
| rs360719 | 32% | | OR=1.30 | 97% |
|  |  |  | OR=1.20 | 76% |
|  |  |  | OR=1.10 | 28% |

The estimation was performed considering an average of Chagas disease prevalence in Argentina and Colombia: 2.52%. MAF: minor allele frequency. The allele frequencies used were those described for the Americans sub-populations of the 1000 genomes phase III project (<http://www.1000genomes.org>).

S1-B2 Table

|  |  |  | *Argentina, Colombia, Bolivia, Brazil* |
| --- | --- | --- | --- |
| SNP | MAF | OR | Chronic Chagas cardiomyopathy (n=1707/1183)* |
| rs2043055 | 41% | OR=1.30 | 100% |
|  |  | OR=1.20 | 94% |
|  |  | OR=1.10 | 45% |

The estimation was performed considering an average of Chagas disease prevalence in Argentina, Colombia, Bolivia and Brazil: 3.385%. MAF: minor allele frequency. The allele frequency used were those described for the Americans sub-populations of the 1000 genomes phase III project (<http://www.1000genomes.org>).

S1-B3 Table

|  |  |  | *Argentina, Colombia, Bolivia* |
| --- | --- | --- | --- |
| SNP | MAF | OR | Chronic Chagasic cardiomyopathy (n= 858/981)* |
| rs1946518 | 49% | OR=1.30 | 99% |
|  |  | OR=1.20 | 82% |
|  |  | OR=1.10 | 32% |
| rs360719 | 32% | OR=1.30 | 98% |
|  |  | OR=1.20 | 78% |
|  |  | OR=1.10 | 29% |

The estimation was performed considering an average of Chagas disease prevalence in Argentina, Colombia and Bolivia: 3.713%. MAF: minor allele frequency. The allele frequencies used were those described for the Americans sub-populations of the 1000 genomes phase III project (<http://www.1000genomes.org>).

*Analysis performed by using cases vs. controls individuals.

**Bibliography**

[1]. Word Health Organization/Department of control of neglected tropical diseases (2017) Integrating neglected tropical diseases in global health and development. 4th WHO report on neglect trop diseases. Geneva: World Health Organization.

[2]. Dias JC, Ramos AN Jr, Gontijo ED, Luquetti A, Shikanai-Yasuda MA, et al. (2016) Brazilian Consensus on Chagas Disease, 2015. Epidemiol Serv Saude 25: 7-86. doi: 10.5123/S1679-49742016000500002. PMID: 27869914
