# Supplementary material for: Association of IL18 genetic polymorphisms with Chagas disease in Latin American populations
Source: PLoS Negl Trop Dis. 2019 Nov 21;13(11):e0007859. doi: 10.1371/journal.pntd.0007859 (PMC6894881; doi:10.1371/journal.pntd.0007859)
Supplement: S2 Table — In silico functional characterization of IL18 gene variants (A) Regulatory feature consequences for IL18 gene variants (B). (DOCX) [file pntd.0007859.s003.docx]

**S2-A Table. Functional annotation. Regulatory chromatin states and histone modifications for *IL18* gene variants**

| **Position^a^** | **SNPs** | **Functionality** | **Description tissue type** | **Chromatin states^b^** | **H3K4me3** | **H3K27ac** | **H3K9ac** |  |
| --- | --- | --- | --- | --- | --- | --- | --- | --- |
| chr11:112031624 | rs2043055 | Intron | Primary mononuclear cells from peripheral blood | Enhancer | - | - | - |  |
| chr11:112035458 | rs1946518 | Intron | Primary mononuclear cells from peripheral blood | - | Promoter | Enhancer | Promoter |  |
|  |  |  | Primary T helper naive cells from peripheral blood | - | - | - | Promoter |  |
| chr11:112036149 | rs360719 | Intron | Primary mononuclear cells from peripheral blood | - | Promoter | - | Promoter |  |
|  |  |  | Primary T helper naive cells from peripheral blood | - | - | - | Promoter |  |

^a^ According to National Center for Biotechnology Genome Reference Consortium NCBI build GRCh37

^b^Chromatin states: 25-state model using 12 imputed marks.

H3K4me1: Histone H3 lysine 4 mono-methylation, H3K4me3: Histone H3 lysine 4 tri-methylation, H3K27ac: Histone H3 lysine 27 acetylation, H3K9ac: Histone H3 lysine 9 acetylation

**S2-B Table. Regulatory feature consequences for *IL18* gene variants**

| **SNPs** | **Active in cells lines^a^** | **Feature type^a^** | **Consequence type^a^** | **Regulatory regions*** | **Cell type*** |
| --- | --- | --- | --- | --- | --- |
| rs2043055 | Monocytes-CD14+, neutrophil, CD14+CD16- monocyte, M1 macrophage, macrophage, neutrophil myelocyte, neutrophil, A549, NHEK, HeLa-S3, NH-A, HSMMtube | Promoter flanking region | Regulatory region variant | Transcription factor | Immortalized human fetal osteoblastic cell line (hFOB) |
| rs1946518 | - | - | - | Transcription factor | Vertebral cancer of the prostate (Vcap)  Pancreatic cancer cell (Pdac) |
| rs360719 | - | - | - | Transcription factor | Pancreatic cancer cell (Pdac) |

^a^ According to Ensembl Browser - Genes and regulation

*According to Remap2018 v1.2 Collection of regulatory regions in human deriving from a large-scale integrative analysis of ChIP-seq experiments for hundreds of transcriptional regulators (TRs) such as transcription factors, transcriptional co-activators and chromatin regulators.
