# Supplementary material for: Association of IL18 genetic polymorphisms with Chagas disease in Latin American populations
Source: PLoS Negl Trop Dis. 2019 Nov 21;13(11):e0007859. doi: 10.1371/journal.pntd.0007859 (PMC6894881; doi:10.1371/journal.pntd.0007859)
Supplement: S1 Membership — (DOCX) [file pntd.0007859.s004.docx]

**Members of Chagas Genetics CYTED Network**

Patricia A. Paglini^1^, Alejandro G. Schijman^2^, Luis E. Echeverría^3^, Manuel Fresno^4^, Maria Jesus Pinazo^5^, Israel Molina^6^, Gilberto Vargas-Alarcón^7^, José E. Calzada^8^, Carlos Robello^9^, Mercedes Fernández-Mestre^10^, Clara I. González^11^, Javier Martín^12^.

1. Centro de Estudios e Investigación de la Enfermedad de Chagas y Leishmaniasis, FCM, INICSA-CONICET-UNC, Córdoba, Argentina

2. Laboratorio de Biología Molecular de la Enfermedad de Chagas, INGEBI-CONICET, Buenos Aires, Argentina

3. Heart Failure and Heart Transplant Clinic, Fundacion Cardiovascular de Colombia, Floridablanca, Colombia

4. Department of Molecular Biology, Centro de Biología Molecular Severo Ochoa (CSIC-UAM), Universidad Autónoma de Madrid, Madrid, España

5. ISGlobal, Hospital Clínic, Universitat de Barcelona , Barcelona , España

6. Unidad de Medicina Tropical y Salud Internacional Hospital Universitari Vall d'Hebron, PROSICS, Barcelona, España

7. Department of Molecular Biology, Instituto Nacional de Cardiologia Ignacio Chavez, Mexico City, Mexico

8. Instituto Conmemorativo Gorgas de Estudios de la Salud (ICGES), Panama City, Panama

9. Laboratory of Host Pathogen Interactions-UBM, Institut Pasteur de Montevideo, Montevideo, Uruguay

10. Laboratorio de Fisiopatología, Centro de Medicina Experimental, Instituto Venezolano de Investigaciones Científicas, Caracas 21827, Venezuela

11. GIEM, Universidad Industrial de Santander, Bucaramanga, Colombia

12. Instituto de Parasitología y Biomedicina López-Neyra, IPBLN-CSIC, PTS Granada, Granada, España
